# Application of a new integrated low-profile anterior plate and cage system in single-level cervical spondylosis: a preliminary retrospective study

**DOI:** 10.1186/s13018-022-02917-9

**Published:** 2022-01-15

**Authors:** Leixin Wei, Chen Xu, Minjie Dong, Yibo Dou, Ye Tian, Huiqiao Wu, Xiaodong Wu, Xinwei Wang, Huajiang Chen, Xiaolong Shen, Peng Cao, Wen Yuan

**Affiliations:** Department of Orthopedics, Shanghai Changzheng Hospital, Naval Medical University, 415th Fengyang Road, Shanghai, 200003 China

**Keywords:** Cervical spondylosis, Alignment, ACDF, Carmen

## Abstract

**Background:**

Although ACDF has been widely used in treating cervical spondylosis and related diseases, the complications along with this anterior surgical technique have hindered its application and affected the postoperative outcome of the patients. Here, we investigated the clinical and radiological outcomes of a new integrated low-profile anterior plate and cage system for anterior cervical discectomy and fusion (ACDF) in treating cervical spondylosis.

**Methods:**

A total of 96 cervical spondylosis patients who underwent single-level ACDF between 2018 to 2020 in our institute were enrolled. There were 28 patients using the new implants and 68 patients using the zero-profile (Zero-P) implants. The Japanese Orthopedic Association (JOA) score and the visual analog scale (VAS) were used to evaluate the clinical outcomes. The cervical and segmental Cobb angle and range of motion (ROM) were used to assessed the radiological outcomes. Incidence of complications were also recorded. All data were recorded at pre-operation, 6-month and 12-month post-operation.

**Results:**

All patients were followed-up for at least 1-year, the mean follow-up time was over one year. The fusion rate was similar in the two groups. There was no significant difference in the postoperative JOA score recovery rate, postoperative VAS score of neck and arm pain, postoperative ROM, and incidence of complications between two groups (*P* > 0.05). However, postoperative cervical and segmental Cobb angle were better maintained in the new low-profile implant group compared to Zero-P group.

**Conclusions:**

The clinical outcomes of the new low-profile implant were satisfactory and comparable to that of zero-profile system. It may have advantages in improving and maintaining the cervical lordosis, and can be an alternative device for single-level cervical spondylosis treated with ACDF.

## Background

Anterior Cervical Discectomy and Fusion (ACDF) is one of the most common and effective surgical approach to treat cervical spondylosis. Since first introduced by Smith Robinson [1] and Cloward [2] in 1958, the ACDF procedure has become widely used till nowadays and is still proved as a standard procedure for cervical spondylosis. Although the surgical approach has not been changed for many years, the implants on the other hand have kept involving to minimizing the side effects and complications along with this surgical method.

**Fig. 1:**
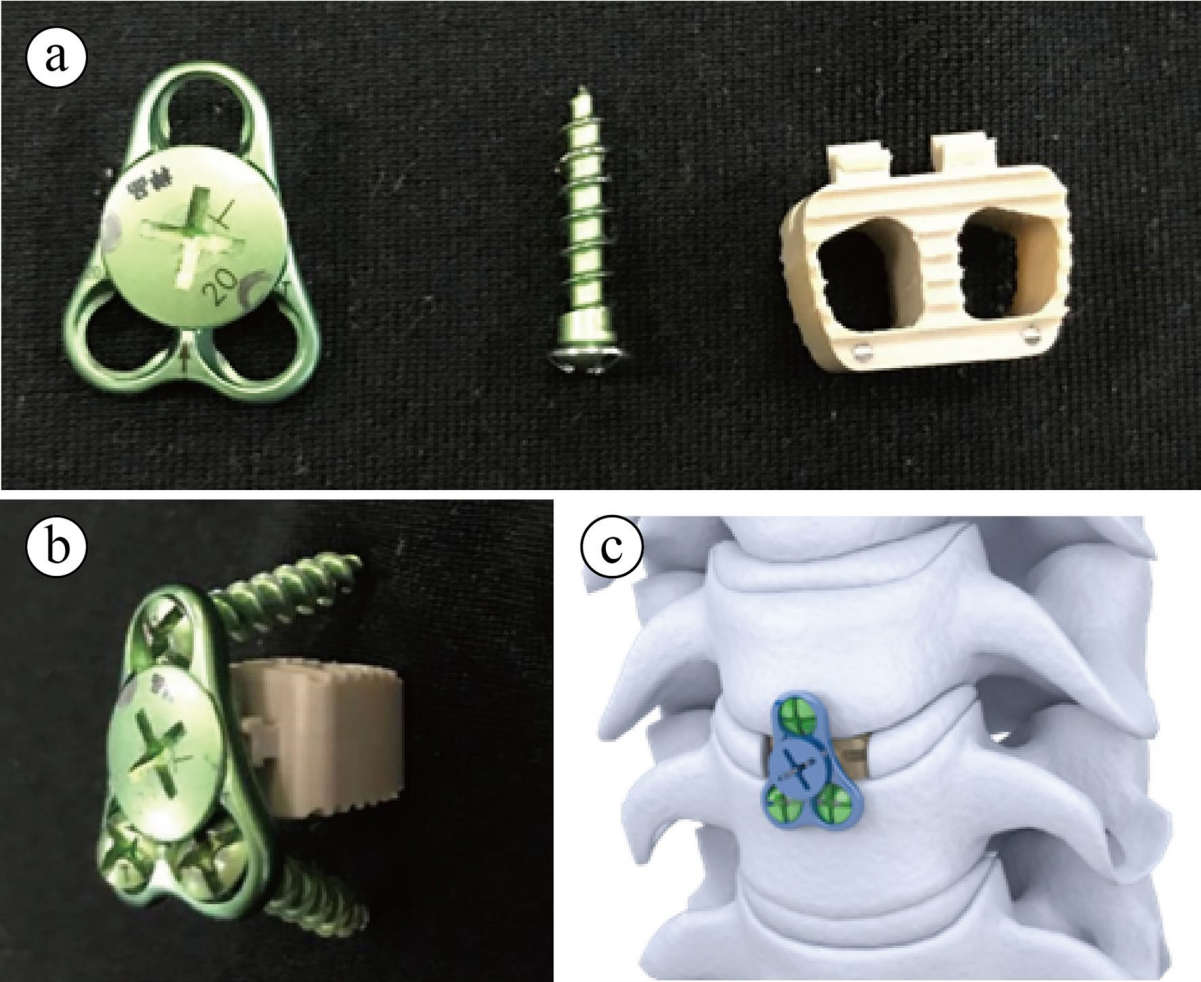
The design of a new integrated low-profile mini plate and cage system. **a** The new integrated low-profile mini plate and cage system can be divided into a triangular plate piece, the locking screw and self-drilling screw, and a polyetheretherketone (PEEK) cage with hook to attach to the plate (**b**). When used in cervical ACDF surgery, only the thin triangular titanium plate protrudes the vertebrate (**c**)

Varies implants were designed to promote segmental fusion and achieve better clinical outcomes. Although traditional bone grafting is reliable, It is then replaced by plate and cage system for its stability and does not need to prepare grafting materials from iliac bone of the patients. However, despite its advantages at restoring cervical alignment and maintain cervical height, it may also lead to complications like dysphagia, esophageal perforation and adjacent segment degeneration [3–5]. Recently, the zero-profile implant (Zero-P) has been invented and widely used for one or two segmental ACDF surgeries [6], and found that Zero-P implant could attain similar clinical results and significantly lowered the incidence of dysphagia and adjacent segment degeneration when compared to plate and cage system [7–11]. The reason of which may be due to its integrated design that does not protrudes the front rim of the cervical vertebrate. It is not until recent years that studies have reported that postoperative loss of cervical alignment has been observed in ACDF with Zero-P implant [12, 13], and further caused recurrent symptoms or revisions. Compared to titanium plate and cage system, Zero-P implant may cause postoperative lordosis loss and bone absorption, which is more significant on longer terms [13]. Considering the drawbacks of both types of implants, we here present a new integrated low-profile anterior plate and cage system (Carmen, Shanghai Sanyou Medical Co., Ltd, Shanghai, China) which is approved by the United States Food and Drug Administration in 2018. In this system, a titanium triangular thin plate with 3 screw trajectories can be attached to polyetheretherketone (PEEK) made disc spacer (Fig. 1a–c), since it mimics the zero-profile system and retain small plate design for screw fixation, we name it a low-profile anterior plate and cage system. The system is designed to combine both the advantages of traditional plate and cage system and zero-profile implants to avoid the complications in either system. Thus, the purpose of this study is to give the initial outcome of this new system and compare the clinical and radiological outcomes with the zero-profile implant.

## Methods

### Patient population

All study procedures were approved by the institute chancellor’s Human Research Committee in accordance with institute’s protocol. Patients who underwent single-level anterior cervical diskectomy and fusion (ACDF) for diagnosed cervical radiculopathy or myelopathy between January 2018 and January 2020 in our institute were reviewed. The exclusion criteria included as follows: (1) ossification of the posterior longitudinal ligament; (2) acute spinal cord injuries; (3) severe cervical kyphosis; (4) history of cervical surgery; (5) thoracic or lumbar diseases; (6) history of rheumatoid, cerebral palsy, or tumors. Altogether 28 patients who underwent single-level ACDF with the new low-profile anterior plate and cage system (Carmen, Shanghai Sanyou Medical Co., Ltd, Shanghai, China) is included in this study, and 68 patients with Zero-P (DePuy Synthes, USA) for single-level ACDF symptomatic cervical spondylosis were retrospectively enrolled in this study. All patients were followed up regularly, with the mean follow-up time of 15.19 months (from 12 to 24).

### Surgical technique

Under general anesthesia, the patients were placed in supine position. Then, a standard Smith-Robinson method was used. The surgical level was confirmed by intraoperative radiography. A thorough decompression was performed by removing the disc, part of posterior longitudinal ligament, and osteophytes. After scraping off the cartilaginous endplate, a suitable Carmen implant (Carmen group, Fig. 1a–c) or Zero-P implant (Zero-P group) was inserted in the disc space. And correct position of implants was reconfirmed by using intraoperative X-ray. All the ACDF surgeries were performed by the same surgeon. Patients were allowed to sit up and walk on the second day after surgery with a protection of Philadelphia collars. The collars were maintained in 2–3 weeks for all patients.

### Clinical outcomes

Intraoperative measurements, including operative time and blood loss, were recorded. The clinical outcomes were assessed by using the Japanese Orthopedic Association (JOA) score and visual analog scale (VAS) of neck and arm pain. The recovery rate of JOA score was calculated by the following formula: Recovery rate (%) = (postoperative JOA score − preoperative JOA score)/(17 − preoperative JOA score) × 100%. Besides, incidence of dysphagia was also recorded.

### Radiographical outcomes

All the patients were performed anteroposterior, lateral, and maximal flexion–extension lateral radiographic images before and after surgery. All the radiological measurements were analyzed by the Image J software (National Institutes of Health, Bethesda, Maryland).

The cervical alignment assessed by Cobb angle was measured between the upper endplate of C2 and the lower endplate of C7. Similarly, the segmental lordosis of the surgical level was measured by using Cobb method between the upper endplate of the upper vertebrate body and the lower endplate of the lower vertebrate body. Range of motion (ROM) of cervical spine was also measured by calculating the difference in the alignment at flexion and extension [14]. Proportion of ROM preserved (ROM preservation) was calculated by the following formula: ROM preservation = (postoperative ROM)/(preoperative ROM) × 100%.

### Statistical analysis

The data in this study were collected and analyzed by using the SPSS 18.0 (SPSS, Inc., USA). Continuous variables were shown in means and standard deviations (SD), and categorical variables in frequencies and percentages. Continuous variables were first tested for normality using the Shapiro–Wilk test, and statistically significant differences between the different subgroups were tested with Pearson’s Chi-square tests for categorical and Mann–Whitney U tests for continuous data that did not passed the normality test, otherwise were tested with Student’s t-test. *P* value less than 0.05 was considered statistically significant.

## Results

### Patients’ demographic analysis

Demographic data were summarized in Table 1. The new integrated low-profile implant Carmen was first used in 2018 in our institute, and was used in 28 patients until January 2020 (all patients were diagnosed as single-level cervical spondylosis, and were defined as Carmen group in the following study). 68 patients who received single-level ACDF with Zero-P device were defined as Zero-P group. The surgical segments ranged from C3 to C7, and the distribution of lesion segment showed no significant difference between two groups (*P* = 0.208). There were also no statistic differences in the gender, age, smoking status, BMI and duration of symptoms between two groups (*P* > 0.05, Table 1).

**Table 1:** Demographic data of study groups

	Carmen group	Zero-P group	*P* value
Patients (*n*)	28	68	
*Gender*			0.400
Male	15	36	
Female	13	32	
Age (years)	47.2 ± 6.7	48.7 ± 7.3	0.339
*Smoking status*			0.957
Smoking	12	30	
Non-smoking	16	38	
BMI	22.5 ± 1.8	22.8 ± 2.2	0.488
Duration of symptoms (months)	14.4 ± 7.6	15.4 ± 5.9	0.492
*Lesion segment*			0.208
C3/4	5	6	
C4/5	8	21	
C5/6	15	32	
C6/7	9	9	
Follow-up time	15.1 ± 5.2	15.3 ± 5.2	0.859

BMI, body mass index. A *P* value of less than 0.05 was considered to indicate a statistically significant difference

### Neurological outcome

The mean operative time, blood loss, and fusion rate were similar between two groups (Table 2). The mean JOA score was 9.76 ± 0.76 preoperatively and 13.58 ± 1.59 (6 months), 15.28 ± 1.17 (12 months) postoperatively, with the mean recovery rate of 76.24 ± 7.24 in the Carmen group at 12-month follow-up. And similar result was found in Zero-P group, with the mean JOA score was 9.52 ± 0.81 preoperatively and 13.12 ± 1.67 (6 months), 14.52 ± 1.23 (12 months) postoperatively, and the mean recovery rate was 66.84 ± 7.96 at 12-month follow-up. With regard to neck and arm VAS outcome, we also found that no significant differences exist between the two groups (*P* > 0.05, Table 2).

**Table 2:** Clinical outcomes between two implant groups

	Carmen group	Zero-P group	*P* value
Operative time (min)	53.16 ± 5.29	55.24 ± 5.17	0.082
Blood loss (mL)	32.46 ± 8.31	34.52 ± 6.42	0.218
Fusion rate (%)	100	98.5	0.921
*JOA score*			
Preoperation	9.76 ± 0.76	9.52 ± 0.81	0.442
Postoperation (6 m)	13.58 ± 1.59	13.12 ± 1.67	0.298
Postoperation (12 m)	15.28 ± 1.17	14.52 ± 1.23	0.213
Recovery rate (6 m)	52.76 ± 7.81	48.13 ± 8.02	0.141
Recovery rate (12 m)	76.24 ± 7.24	66.84 ± 7.96	0.064
*Neck VAS*			
Preoperation	4.16 ± 0.80	4.43 ± 0.88	0.452
Postoperation (6 m)	0.86 ± 0.75	1.02 ± 0.78	0.137
Postoperation (12 m)	0.52 ± 0.43	0.89 ± 0.51	0.052
*Arm VAS*			
Preoperation	6.70 ± 0.87	6.81 ± 0.89	0.573
Postoperation (6 m)	0.68 ± 0.71	0.81 ± 0.81	0.223
Postoperation (12 m)	0.55 ± 0.40	0.77 ± 0.52	0.134

JOA, the Japanese Orthopedic Association score; VAS, the visual analog scale. A *P* value of less than 0.05 was considered to indicate a statistically significant difference

### Radiological outcome

**Fig. 2:**
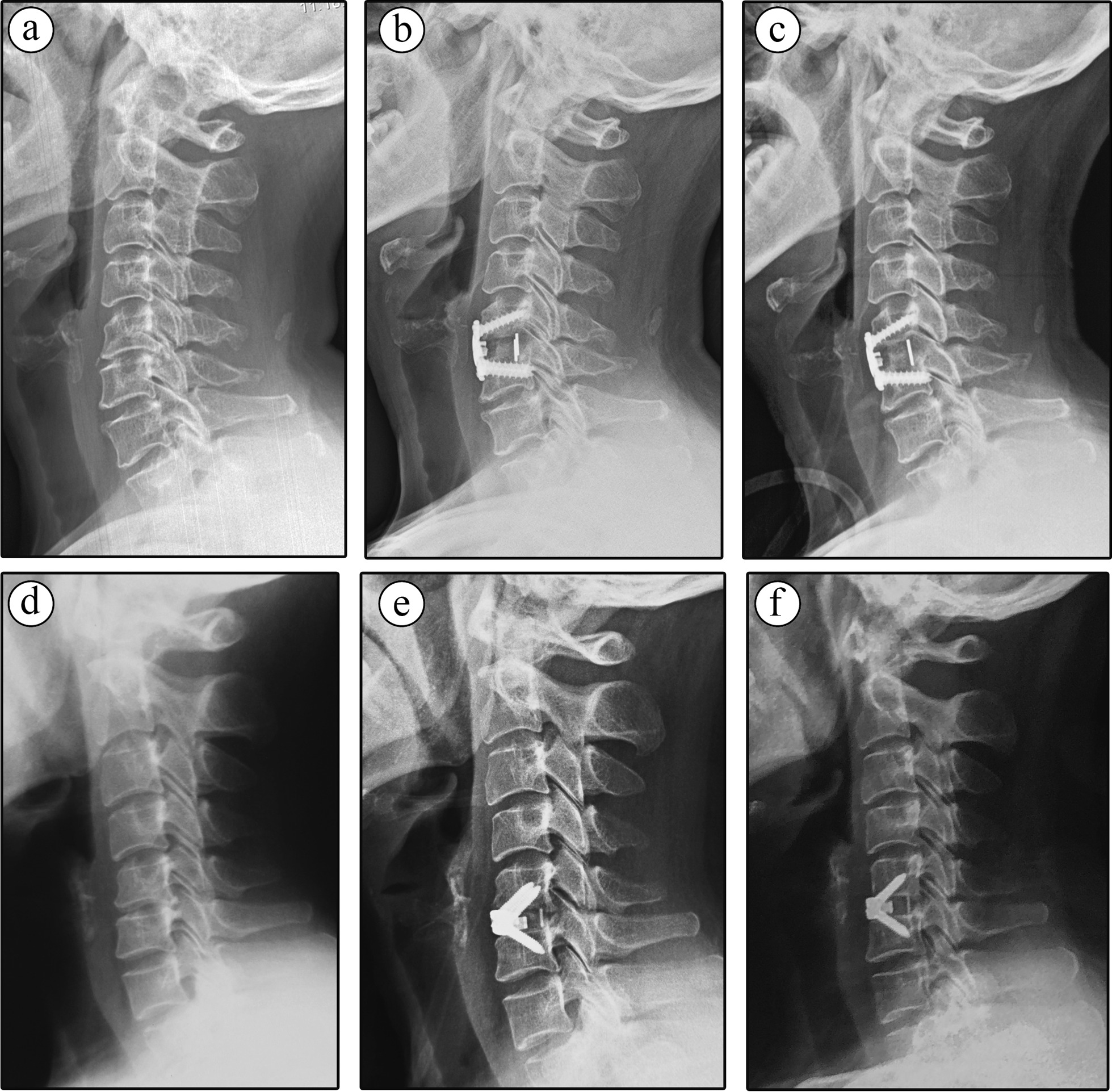
Representative cervical X-ray images of Carmen and Zero-P implant treated patient. **a**–**c** Single-level male patient treated with Carmen device, the segmental Cobb angle of C5–C6 changed from 11-degree lordosis to 18-degree lordosis after the surgery, and reached 20-degree at one-year follow-up, as well as the C2–C7 Cobb angle. **d**, **e** Single-level male patient treated with Zero-P device, the segmental Cobb angle of C5–C6 changed from 7-degree lordosis to 9-degree lordosis after the surgery, and decreased to 4-degree at one-year follow-up, as well as the C2–C7 Cobb angle (15-degree preoperative, 20-degree postoperative and 15-degree at one-year follow-up)

Cervical alignment and the segmental Cobb angle of the surgery level were assessed and compared between the two groups. Although no significant difference was found in pre-operative cervical Cobb angle and segmental Cobb angle between two groups (*P* > 0.05), and initial post-operative (3 days P.O.) cervical Cobb angle and segmental Cobb angle between two groups (*P* > 0.05), both the cervical Cobb angle and segmental Cobb angle showed significant differences at one-year follow-up (Table 3), with the cervical Cobb angle and segmental Cobb angle significantly higher in the Carmen group than that in the Zero-P group (*P* < 0.01, Fig. 2a–f). Besides, there was no statistic difference in preoperative ROM, postoperative ROM, and its preservation between two groups (*P* > 0.05). Figure 2 shown the representative cervical X-ray images of typical case from the Carmen group and the Zero-P group, respectively.

**Table 3:** Radiological features between two implant groups

	Carmen group	Zero-P group	*P* value
*Cervical Cobb angle (°)*			
Preoperation	8.72 ± 5.82	9.35 ± 4.82	0.453
Postoperation (3 days)	16.19 ± 4.95	15.33 ± 4.42	0.405
Postoperation (12 m)	15.98 ± 5.25	12.53 ± 4.42	0.012*
*Segmental Cobb angle (°)*			
Preoperation	3.01 ± 1.33	3.42 ± 1.25	0.174
Postoperation (3 days)	5.59 ± 1.76	5.73 ± 1.57	0.702
Postoperation (12 m)	5.69 ± 1.71	4.19 ± 1.18	0.024*
*Total ROM (°)*			
Preoperation	38.31 ± 7.49	37.22 ± 6.79	0.538
Postoperation (12 m)	33.25 ± 7.43	31.48 ± 6.91	0.242
Preservation rate (%)	87.53 ± 15.89	85.07 ± 14.92	0.321

ROM, range of motion. A *P* value of less than 0.05 was considered to indicate a statistically significant difference

### Complications

The incidence of postoperative dysphagia occurred in 1 patient in Carmen group and 2 in Zero-P group, all these patients had no symptom at 6-month follow-up, and no patient suffered severe dysphagia (Table 4). For adjacent segment degeneration, 2 patients in Carmen group and 5 patients in Zero-P group had adjacent disc degeneration during follow-up, but no significant compression and neurological symptoms occurred (Table 4). 2 patients in Zero-P group suffered postoperative axial pain after the surgery, and recovered within 6-month (Table 4). 1 patient had pseudoarthrosis in Zero-P group and did not achieve fusion until 12-month post-operation (Table 4).

**Table 4:** Incidence of complications between two groups

	Carmen group	Zero-P group	*P* value
Dysphagia	1 (3.6)	2 (3.0)	0.872
ASD (n, %)	2 (7.1)	5 (7.4)	0.971
Axial pain (n, %)	0 (0)	2 (3.0)	0.359
Pseudoarthrosis	0 (0)	1 (1.5)	0.519
Implant failure	0 (0)	0 (0)	–
Revisions	0 (0)	0 (0)	–

ASD, adjacent segment degeneration. A *P* value of less than 0.05 was considered to indicate a statistically significant difference

## Discussion

Since ACDF was first introduced by Smith Robinson and Cloward in 1958, this surgical technique has been widely used for the treatment of cervical degenerative disc diseases [1]. Initially, autologous bone graft was used to achieve intervertebral fusion. However, the use of bone graft could lead to the donor-site complications including hematoma formation, neurological injury, infection, and pain [15]. Later on, various types of implants and devices have been developed to assist intervertebral fusion. Of all devices, the plate and cage system can provide solid stability and improve cervical sagittal alignment [16], and is widely used till now due to such advantages. However, this system has been reported to have higher risks of having complications like dysphagia and adjacent segment degeneration (ASD), which were not negligible. Thus, recently developed zero-profile implant system (Zero-P for example) was designed to decreased the incidence of dysphagia and ASDs [17].

Although Zero-P system has been proved to have advantages over traditional plate and cage system in postoperative complications, and has comparable clinical outcomes to traditional plate and cage system as provided by previous researches [7–9, 11, 18], it is less efficient in dealing with cervical alignment anomalies. Recent reports have demonstrated that compared with titanium plate and cage system, zero profile system failed to maintain C2-7 Cobb angle, segmental Cobb angle and adjacent vertebral height in longer follow-ups [16]. While other reports showed that multiple Zero-P fixation failed to maintain cervical lordosis when compared to plate and cage system [13]. And recent meta-analysis found that traditional plate and cage system can restore the cervical alignment better than Zero-P implants [10, 12, 19, 20]. Thus, how to achieve best clinical outcomes and prevent loss of lordosis and other complications like ASDs after ACDF is still controversial.

Here, we retrospectively studied the a newly designed low-profile mini plate system, the Carmen system, and compared the initial clinical outcome and radiological results after one-year follow-up in single level cervical spondylosis. The findings showed that, both the Carmen system and Zero-P system could achieve similar neurological outcome and maintain functional recovery till one-year follow-up, which confirmed that the Carmen design is eligible for ACDF surgeries. Cervical lordosis plays an important role in maintaining the sagittal alignment and spinal balance. Loss of cervical lordosis is related to pain, disability, and undesirable loading shift of the thoracic and lumbar spine, and could result in adjacent segment degeneration or other issues [7]. For Carmen is mainly aimed to achieve better lordosis maintenance than the zero-profile system. The plate of which is designed with low profile triangle shape, which automatically affixes the bone surface and accords with the direction of vertical stress conduction. The triangular frame can effectively control the transverse shear force caused by fretting, ensure fusion, and avoid postoperative complications. Here we found that both the C2–C7 cervical Cobb and segmental Cobb angle were initially restored after the surgery, and were maintained during follow-ups in the Carmen group than those in the Zero-P group. The possible reason for these results may be due to the trilateral mini plate designed to restrict cage subsidence and maintain segmental curvature. Another possible reason may be attributed to the screw placement differences in two devices, which they go through the cortical bone in a more parallel angle in Carmen implant, while the screws go through the cage first and then go through the endplate to the cancellous bone in zero profile device, as mentioned in previous studies [16, 21].

Dysphagia is a well-known postoperative complication after ACDF. The incidence of dysphagia varies widely, with the rate of 50.2%, 32.2%, 17.8%, and 12.5% at 1, 2, 6, and 12 months, respectively [22]. The exact reason for dysphagia is still unclear. Female gender, multiple surgical level, esophageal injury, postoperative soft tissue edema, adhesive formations around implanted cervical plates, and postoperative hematoma may be the risk factors for dysphagia. Previous studies reported that the incidence of dysphagia was significantly higher in traditional plate and cage than that in the Zero-P implant [20, 23]. In this study, we found that the use of the Carmen implant and the Zero-P implant both led to the similar incidence of dysphagia after the operation, and none had severe dysphagia more than 6 months. Lee et al. [24] reported that the use of a smaller and smoother plate indeed reduced the incidence of dysphagia as compared with a slightly larger and less smooth plate. In our new design, the trilateral plate used in Carmen implant is smaller and smoother than traditional plate. So, this is the possible reason for similar incidence of dysphagia in the Carmen implant compared with the Zero-P implant. For other complications like ASDs, axial pain and pseudoarthrosis, both implants showed no differences when compared, and it may be due to the sample size that needs further studies.

The present study also had several limitations. Firstly, the nature of this study was retrospective, the observed results should be further studied using cohort to rule out the bias. Secondly, the patient population was relatively small in the Carmen implant group, for initial data is needed to further expand its usage, which will be done in the future. Thirdly, the follow-up duration was relatively short. Despite these limitations, this study was the first clinical evaluation study of the newly designed Carmen implant for ACDF treated cervical spondylosis patients.

## Conclusions

When compared to the zero-profile implant, the primary clinical outcomes of the integrated low-profile mini plate implant used in ACDF was satisfactory and was comparable to that of zero-profile system. Besides, this implant may have advantages in improving and maintaining the cervical and segmental lordosis compared to zero-profile system. The new integrated low-profile mini plate (Carmen) system can be a better alternative in single-level cervical spondylosis treated with ACDF.

## Data Availability

Data sharing is not applicable to this article as no datasets were generated or analysed during the current study.

## Abbreviations

ACDF: Anterior cervical discectomy and fusion
ASD: Adjacent segment degeneration
Zero-P: Zero-profile
JOA: Japanese Orthopedic Association score
VAS: Visual analog scale
ROM: Range of motion
